# Rapidly Growing and Ruptured Great Saphenous Vein Aneurysm in a Liver Transplant Patient

**DOI:** 10.3390/medicina60020290

**Published:** 2024-02-08

**Authors:** Mark Racman, Jan Kafol, Borut Jug, Milenko Stankovic, Dragan Piljic, Jus Ksela

**Affiliations:** 1 Department of Cardiovascular Surgery, University Medical Centre Ljubljana, 1000 Ljubljana, Slovenia; mark.racman@kclj.si; 2 Faculty of Medicine, University of Ljubljana, 1000 Ljubljana, Slovenia; jan.kafol@gmail.com (J.K.); borut.jug@kclj.si (B.J.); 3 Department of Vascular Diseases, University Medical Centre Ljubljana, 1000 Ljubljana, Slovenia; 4 Clinical Institute of Radiology, University Medical Centre Ljubljana, 1000 Ljubljana, Slovenia; milenko.stankovic@guest.arnes.si; 5 Department of Cardiovascular Surgery, University Clinical Center Tuzla, 75000 Tuzla, Bosnia and Herzegovina; dragan.piljic@dr.com

**Keywords:** venous aneurysm, rapid growth, rupture, immunosuppression, liver transplant

## Abstract

Venous aneurysms are rare vascular malformations that can lead to significant clinical complications, including thrombosis, pulmonary embolism, rupture, and even fatal outcomes when not promptly and adequately managed. This case report presents a liver transplant patient under immunosuppressive therapy who developed a rapidly progressing great saphenous vein aneurysm, ultimately requiring urgent surgical intervention due to acute bleeding from the ruptured aneurysm. Immunosuppression emerges as a potential key factor in the formation and rapid growth of the aneurysm, with the pathophysiological mechanism potentially involving increased expression of specific matrix metalloproteinases. Further research is warranted to gain a better understanding of the role of immunosuppression in the development of venous aneurysms.

## 1. Introduction

Venous aneurysms are rare vascular abnormalities, defined as a localized venous dilation of at least twice the normal vein diameter. In order to be classified as a venous aneurysm, the venous malformation has to connect to a main venous structure through a single channel and must therefore be differentiated from arteriovenous communication or pseudoaneurysm [1]. Venous aneurysms can manifest in all major veins of the human body. Their presence has been reported in the intra- and extra-cranial veins, in the superior vena cava, in the veins of the upper and lower extremities, the common iliac veins, and the spleno-portal system. Depending on the anatomical position of the main venous structure, venous aneurysms of extremities can be classified as either superficial or deep. The exact pathophysiology of their formation is unclear, and several different mechanisms have been suggested [2,3]. Clinical presentation varies, with many cases being asymptomatic and incidentally discovered through imaging. Symptomatic cases may present as painful masses with edema. If not promptly treated, potential severe complications, such as deep vein thrombosis, pulmonary embolism, rupture, and even death may arise [4,5].

Herein, we present a case of a 6.5 cm great saphenous vein aneurysm in a liver transplant patient on a strict immunosuppressive regimen. The aneurysm exhibited rapid growth, leading to a rupture that required urgent surgical intervention. We investigate the potential link between the aneurysm’s origin and its fast progression in the context of immunosuppressive treatment. Patient consent for the case report was obtained.

## 2. Case Presentation

**Figure 1:**
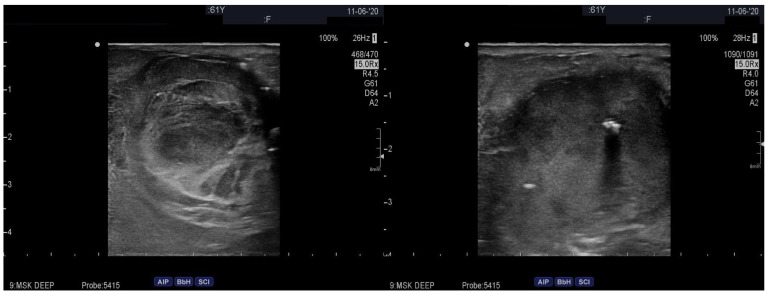
Great saphenous vein aneurysm on initial ultrasound examination.

**Figure 2:**
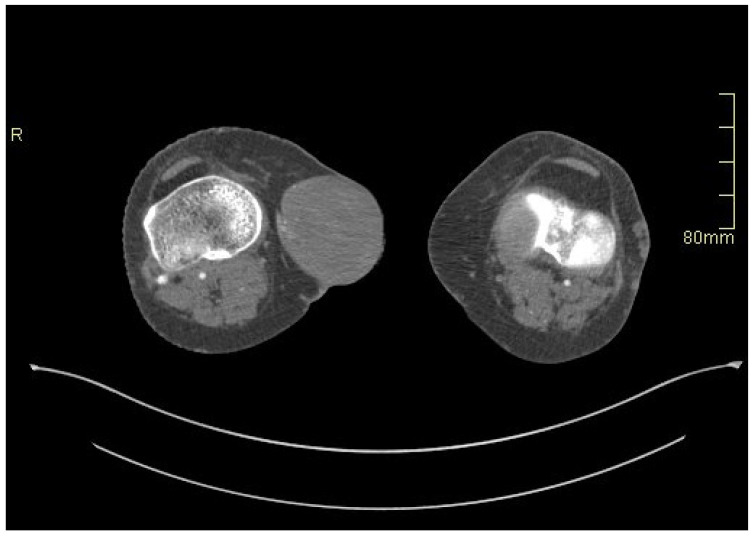
Great saphenous vein aneurysm on CTA.

A 61-year-old Caucasian female liver transplant patient on triple immunosuppressive therapy (i.e., peroral administration of methylprednisolone 4 mg once daily, mycophenolate mofetil 500 mg once daily, and tacrolimus 8 mg once daily; she had been receiving immunosuppressive therapy for about 1 year) and with milder signs of lower extremities’ venous insufficiency, slowly progressing from the pre-transplant period (i.e., from spider veins and some venous nodes pre-transplant to several venous nodes and focal skin discoloration post-transplant, or from the C1/C2 to the C3/C4a stage according to the CEAP classification [6]) first presented to the outpatient transplant clinic due to discomfort and pain in her right knee. On physical examination, a small resistance on the medial side of the right knee was observed, and an ultrasound of the soft tissues of the right leg was performed, revealing a great saphenous vein aneurysm just below the knee joint, measuring approximately 2.5 × 3 cm (Figure 1). Due to its small size, conservative treatment with analgesics and rest was initially pursued. However, 14 days later, the patient was urgently referred to our institution due to acute bleeding from a ruptured venous aneurysm, which had grown substantially over the course of 14 days. During clinical examination, a large vascular formation on the inner side of the right knee was found, with a small bleeding wound at its proximal part. The patient had not been receiving any anticoagulation treatment and denied any trauma or other factors causing the rupture of the vein aneurysm. A whole body computed tomographic angiography (CTA) was immediately performed, which conformed a ruptured great saphenous vein aneurysm measuring 7 × 7 cm (Figure 2) and, at the same time, excluded any other potential aneurysms in the arterial or venous system. In comparison with the initial ultrasonographic imaging conducted 2 weeks prior, the aneurysm showed a significant increase in size as well as a relevant decrease in vascular wall thickness.

**Figure 3:**
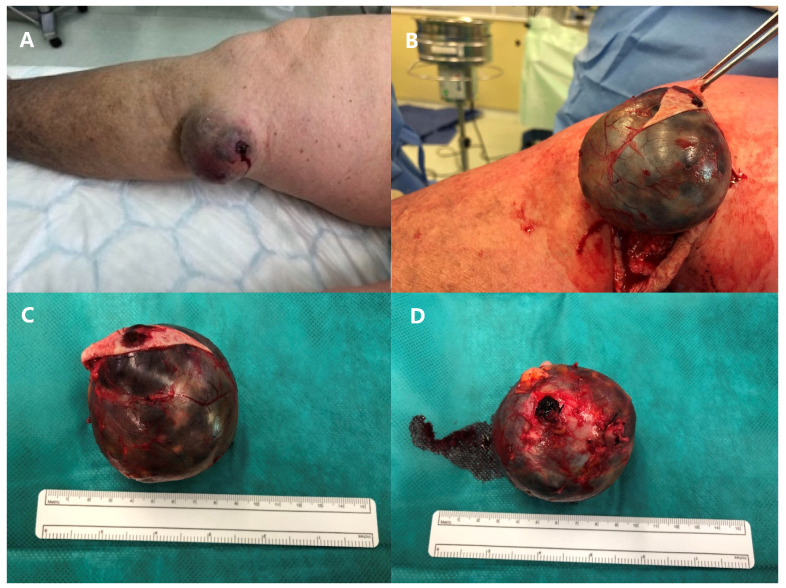
Aneurysm of the great saphenous vein before surgery (**A**), during the surgery (**B**), and after completion of surgical intervention (**C**,**D**).

An urgent surgical resection of the aneurysm was indicated, and access to the vessel was gained through a vertical skin incision on the medial side of the right knee (Figure 3). Both ends of the aneurysm were ligated, and the excised aneurysm was sent for histopathological examination, which later reported a 55 × 65 × 60 mm round, smooth, white-greyish formation filled with blood and with uneven vascular wall thickness (up to 5 mm), with localized wall dissection, and microscopically severe fibro-intimal hyperplasia, focal calcifications, and damage to the elastic layer of the vascular wall. The postoperative course was uneventful, and the patient was discharged on the third postoperative day. Two years after the surgery, the patient remains in stable condition, free from any signs or symptoms of any other vascular aneurysms, and continues to receive outpatient care.

## 3. Discussion

Venous aneurysms represent rare dilations of the veins. They are characterized by a diameter at least 2 times larger than that of adjacent non-dilated veins. They should not be associated with arteriovenous communication, pseudoaneurysms, or varicose veins [7]. Though their first reports date back to 1913, a detailed case series emerged only in 1962 [8,9]. Despite the rarity of this malformation, recent advances in duplex sonography have contributed to an increased number of diagnosed cases [10,11]. The incidence and prevalence of venous aneurysms are equally distributed between genders, and they can develop at any age, although precise epidemiological data are not available [1]. Venous aneurysms can be categorized based on their anatomic location, which significantly influences their clinical importance and determines the treatment strategies [12].

The cause of venous aneurysms is often attributed to trauma, inflammation, infection, venous insufficiency, or hypertension, but many cases remain idiopathic. Spontaneous venous aneurysms can also occur in congenital vascular syndromes, such as Klippel–Trenaunay syndrome [1,3,7].

While the exact pathophysiological mechanisms of venous aneurysm formation and growth remain uncertain, evidence implies that degradation of the extracellular matrix might play a significant role in all vascular aneurysm pathogenesis. Metalloproteinases (MMPs) are remodeling endopeptidases that can degrade most components of the extracellular matrix. They have been demonstrated to have a role in the development of aortic aneurysms and varicose veins, because of their role in tissue homeostasis [13,14]. Elevated concentrations of active MMPs in the aorta result in the degradation of the extracellular matrix. This degradation weakens the aorta’s ability to withstand distending intra-arterial pressure, ultimately leading to the formation of aneurysms [15]. Although the majority of research on the molecular mechanisms of aneurysm development and progression focuses on aneurysms in the arterial system, Irwin et al. evaluated 10 cases of venous aneurysms and demonstrated increased expression of MMP-2, MMP-9, and MMP-13 in endothelial cells, smooth muscle cells, and adventitial micro-vessels of venous aneurysm samples compared to normal vein samples, indicating that MMPs might also play an important role in venous aneurysm pathogenesis [12].

Epidemiological studies have shown increased rates of vascular aneurysm incidence and their faster progression in transplant patients as compared to the healthy population, which is especially true for the incidence of progression and rupture of aortic aneurysms in thoracic and abdominal transplant patients [16]. Although the exact mechanism of aneurysm pathogenesis in this patient population in not completely elucidated, the accumulated evidence suggests that immunosuppression therapy can profoundly disrupt MMPs expression [17,18,19,20]. Sun et al. demonstrated that prednisolone increased the expression of MMP 2, 9, and 13 in the trabecular bone of mice, leading to osteoporosis [17]. Multiple studies have also confirmed that calcineurin inhibitors, such as tacrolimus, which was regularly taken by our patient, can increase MMP 2 and 9 expression, yielding endothelial dysfunction and intimal hyperplasia. However, there are some conflicting reports about the effect of different immunosuppressants on MMPs expression [18,19,20]. We hypothesize that although the pathogenesis of venous aneurysm in our patient was most likely multifactorial, the disturbed MMPs’ metabolism might potentially represent at least one of the determinants leading to the formation and rapid growth of the venous aneurysm in our liver transplant patient who was on a strict triple oral immunosuppressive therapy regime with methylprednisolone, mycophenolate mofetil, and tacrolimus. In this regard, our case is in line with some previous studies implying a possible connection between immunosuppressant generated disturbances of MMPs’ metabolism and vascular wall instability. However, the exact pathophysiological mechanisms connecting immunosuppression therapy and vascular aneurysm formation and development remain unclear, and further research determining the specific molecular pathways leading to vascular wall instability in healthy and transplant populations is warranted. 

Diagnosis of venous aneurysm can be challenging, particularly for saphenous vein aneurysms, as they may mimic soft tissue masses or hernias. The increase in size during an upright position or Valsalva maneuver can be a distinguishing feature, aiding in securing the diagnosis [11,21]. Commonly, the initial presenting symptom of a venous aneurysm is deep vein thrombosis or pulmonary embolism, leading to diagnostic investigations [22]. Among different complications, such as thrombosis or compression of adjacent structures, spontaneous ruptures are the most infrequent; however, they represent a severe and potentially life-threatening complication that necessitates urgent surgical intervention. In contrast to arterial aneurysms, where ruptures are a relatively common complication, there are not many reported cases of ruptured venous aneurysms, which makes our case an unique and interesting clinical scenario [3,5]. In our case, the rupture occurred only about 2 weeks after the initial diagnosis and prompted urgent surgical treatment.

Duplex ultrasonography is the preferred diagnostic tool, given its accessibility, ability to gauge the aneurysm size and thrombus presence, and avoidance of contrast or radiation exposure. Further imaging options include venography, CTA, or MRA [5,23]. Pathological examination of the excised aneurysm can confirm the diagnosis, typically revealing reduced smooth muscle cells and increased fibrous tissue in the vascular wall, resembling the histological structure of arterial aneurysms [1,11,23].

The choice of treatment for venous aneurysms depends on various factors, including the location, size, symptoms, complications, and aesthetic concerns. Due to the rarity of the pathology, there is no consensus on the best treatment approach [1,5,24]. Head and neck venous aneurysms are typically asymptomatic and are treated in approximately half of the cases, often for cosmetic reasons or due to their expansive growth. Thus, the internal jugular vein aneurysms are typically closely followed. Thoracic aneurysms are usually managed conservatively because of their asymptomatic nature. Surgical intervention becomes necessary when there is a significant increase in size. For abdominal aneurysms, surgery is a limited option due to the associated elevated postoperative mortality. Most patients with abdominal aneurysms present with non-specific abdominal pain. Aneurysms in the superficial venous system of upper and lower extremities are often surgically removed. Lower extremity deep venous aneurysms—such as popliteal aneurysms—can lead to pulmonary embolism in the majority of affected patients, and thus, a more aggressive surgical and medical treatment with anticoagulation therapy is warranted [2]. Various surgical approaches for aneurysm removal include ligation, tangential excision with lateral venorrhaphy or autologous vein patch, and complete resection with end-to-end anastomosis or interposition grafting [1,5]. Alternative therapies, such as endovenous laser or radiofrequency ablation, have also been described, but they lack long-term success data [3,23].

After surgery, anticoagulation should be considered for 3–12 months, especially in patients at a higher risk of thrombosis, as was the case in our patient, who received postoperative anticoagulation therapy for 6 months [22].

## 4. Conclusions

Although cases of rapidly growing venous aneurysms and their subsequent rupture are extremely infrequent, we report on a specific clinical scenario of a ruptured rapidly growing great saphenous vein aneurysm in a liver transplant patient. We hypothesize that immunosuppression following transplant surgery may represent a significant determinant of venous vascular wall instability, leading to the development and rapid growth of the venous aneurysm in our patient. Further research is warranted to elucidate the exact pathophysiological mechanisms and molecular pathways involved in venous aneurysm formation and growth to establish optimal treatment strategies and develop suitable follow-up protocols.

## Data Availability

The datasets generated during and/or analyzed during the current study are available from the corresponding author on reasonable request.
